# CT Quantification of COVID-19 Pneumonia at Admission Can Predict Progression to Critical Illness: A Retrospective Multicenter Cohort Study

**DOI:** 10.3389/fmed.2021.689568

**Published:** 2021-06-17

**Authors:** Baoguo Pang, Haijun Li, Qin Liu, Penghui Wu, Tingting Xia, Xiaoxian Zhang, Wenjun Le, Jianyu Li, Lihua Lai, Changxing Ou, Jianjuan Ma, Shuai Liu, Fuling Zhou, Xinlu Wang, Jiaxing Xie, Qingling Zhang, Min Jiang, Yumei Liu, Qingsi Zeng

**Affiliations:** ^1^Department of Radiology, Huangpi District Hospital of Traditional Chinese Medicine, Wuhan, China; ^2^Department of Radiology, Han Kou Hospital of Wuhan, Wuhan, China; ^3^Department of Radiology, The First Affiliated Hospital of Guangzhou Medical University, Guangzhou, China; ^4^Pulmonary and Critical Care Medicine, National Clinical Research Center for Respiratory Disease, National Center for Respiratory Medicine, State Key Laboratory of Respiratory Diseases, Guangzhou Institute of Respiratory Health, The First Affiliated Hospital of Guangzhou Medical University, Guangzhou, China; ^5^Department of Respiratory, First Affiliated Hospital of Guangxi University of Science and Technology, Liuzhou, China; ^6^Department of Pediatric Hematology, Affiliated Hospital of Guizhou Medical University, Guiyang, China; ^7^Department of Hematology, Dawu County People's Hospital, Wuhan, China; ^8^Department of Hematology, Zhongnan Hospital of Wuhan University, Wuhan, China; ^9^Department of Nuclear Medicine, The First Affiliated Hospital of Guangzhou Medical University, Guangzhou, China; ^10^National Clinical Research Center for Respiratory Disease, State Key Laboratory of Respiratory Diseases, Department of Allergy and Clinical Immunology, Guangzhou Institute of Respiratory Health, The First Affiliated Hospital of Guangzhou Medical University, Guangzhou, China; ^11^Department of Pediatrics, The First Affiliated Hospital of Guangxi Medical University, Nanning, China; ^12^Department of Respiratory, Hankou Hospital of Wuhan, Wuhan, China

**Keywords:** COVID-19, pneumonia, critical illness, chest CT, quantification

## Abstract

**Objective:** Early identification of coronavirus disease 2019 (COVID-19) patients with worse outcomes may benefit clinical management of patients. We aimed to quantify pneumonia findings on CT at admission to predict progression to critical illness in COVID-19 patients.

**Methods:** This retrospective study included laboratory-confirmed adult patients with COVID-19. All patients underwent a thin-section chest computed tomography (CT) scans showing evidence of pneumonia. CT images with severe moving artifacts were excluded from analysis. Patients' clinical and laboratory data were collected from medical records. Three quantitative CT features of pneumonia lesions were automatically calculated using a care.ai Intelligent Multi-disciplinary Imaging Diagnosis Platform Intelligent Evaluation System of Chest CT for COVID-19, denoting the percentage of pneumonia volume (PPV), ground-glass opacity volume (PGV), and consolidation volume (PCV). According to Chinese COVID-19 guidelines (trial version 7), patients were divided into noncritical and critical groups. Critical illness was defined as a composite of admission to the intensive care unit, respiratory failure requiring mechanical ventilation, shock, or death. The performance of PPV, PGV, and PCV in discrimination of critical illness was assessed. The correlations between PPV and laboratory variables were assessed by Pearson correlation analysis.

**Results:** A total of 140 patients were included, with mean age of 58.6 years, and 85 (60.7%) were male. Thirty-two (22.9%) patients were critical. Using a cutoff value of 22.6%, the PPV had the highest performance in predicting critical illness, with an area under the curve of 0.868, sensitivity of 81.3%, and specificity of 80.6%. The PPV had moderately positive correlation with neutrophil (%) (*r* = 0.535, *p* < 0.001), erythrocyte sedimentation rate (*r* = 0.567, *p* < 0.001), d-Dimer (*r* = 0.444, *p* < 0.001), high-sensitivity C-reactive protein (*r* = 0.495, *p* < 0.001), aspartate aminotransferase (*r* = 0.410, *p* < 0.001), lactate dehydrogenase (*r* = 0.644, *p* < 0.001), and urea nitrogen (*r* = 0.439, *p* < 0.001), whereas the PPV had moderately negative correlation with lymphocyte (%) (*r* = −0.535, *p* < 0.001).

**Conclusions:** Pneumonia volume quantified on initial CT can non-invasively predict the progression to critical illness in advance, which serve as a prognostic marker of COVID-19.

## Introduction

The rapid spread of coronavirus disease 2019 (COVID-19) has been a global pandemic and a major and urgent threat to the health care system worldwide (1). More than 100 million cases have been reported globally (2). Most COVID-19 patients had mild symptoms of respiratory infection, such as fever and dry cough, but some patients could rapidly develop fatal complications, including respiratory failure requiring mechanical ventilation, septic shock, or even death (3). Until now, no specific treatment strategies have been used in dealing with COVID-19 (4); thus, it is of great importance to predict COVID-19 with worse outcomes, which would enable the introduction of timely treatment and reduce the mortality of patients.

Chest computed tomography (CT) can play a valuable role in screening, diagnosis, and follow-up of COVID-19 patients (5). However, chest CT images are usually interpreted by radiologists, which is subjective with large interobserver and intraobserver variability and thus unable to accurately and quantitatively evaluate the disease severity and is also time-consuming and inefficient (6). Recently, radiomics use a variety of mathematical methods to convert chest CT images into a huge number of minable high-dimensional handcrafted features for predicting prognosis or outcome of COVID-19 patients (7–17). Radiomic features can be used as surrogate biomarkers for biological disease traits such as morphology and heterogeneity. The combination of clinical characteristics and radiomic features from CT could achieve better accuracy in prediction (18, 19). Some studies also apply deep learning to automatically learn features from CT images or in combination with clinical data and radiomics for risk assessment of COVID-19 (20–34). Deep learning and radiomics can be a more objective, quantitative, and stable system for the assessment of the COVID-19 disease course.

The interpretation of quantitative CT features is of great importance for understanding their potential biological meaning. Several biomarkers identified from laboratory features have been used to assess the probability of progressing to severe state in COVID-19 patients (35–38). Therefore, we aimed to investigate the prognostic value of quantitative CT features in predicting the occurrence of critical illness in patients with COVID-19 and the correlation with laboratory features.

## Materials and Methods

### Patient Cohort

This retrospective study was approved by the ethics committee of our hospitals, and the requirement for informed consent was waived. We included COVID-19 patients who admitted to three designated hospitals from December 31, 2019, to March 31, 2020.

**Figure 1:**
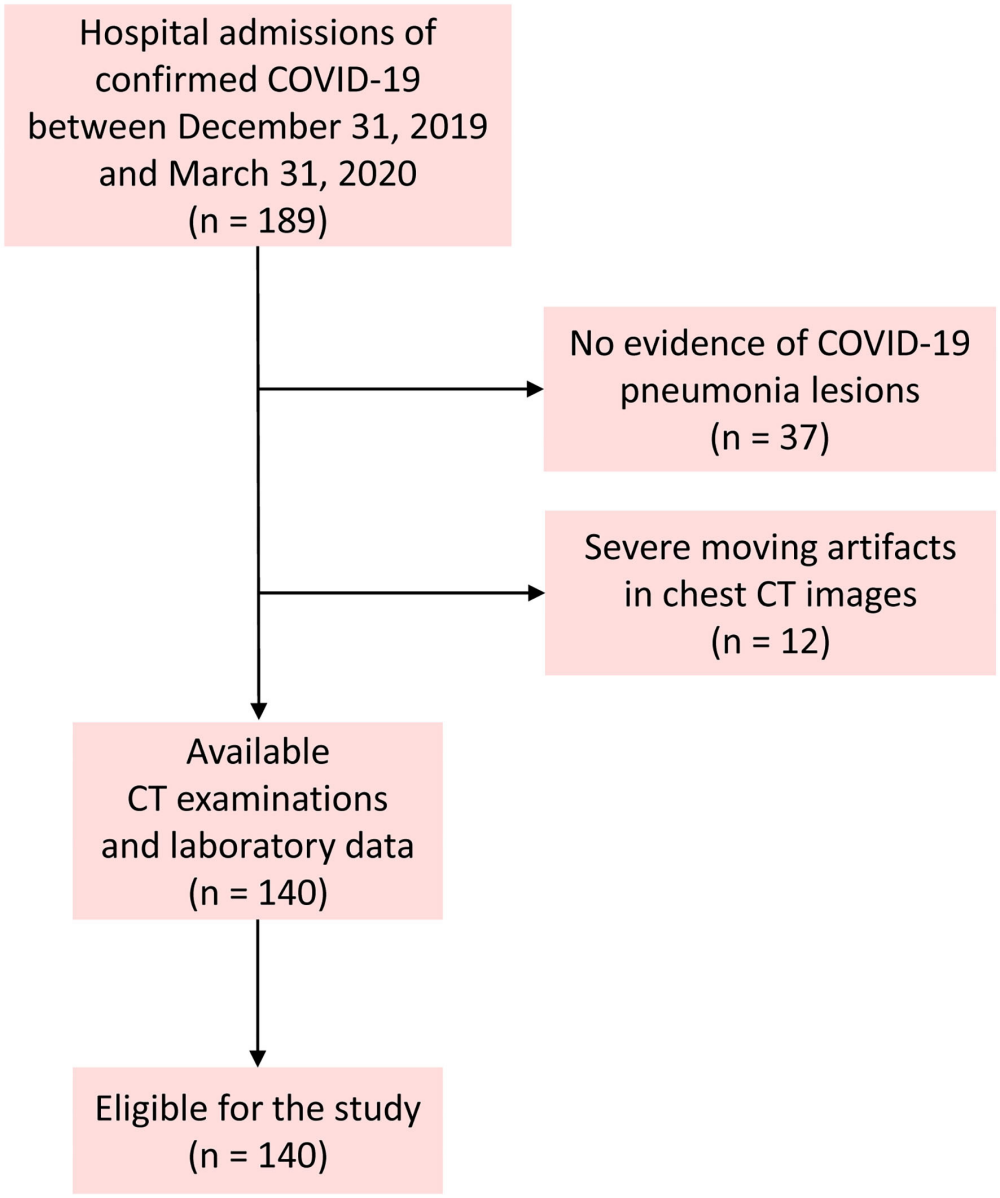
Flowchart of the patient inclusion.

The inclusion criteria were as follows: (1) adult patients; (2) positive real-time reverse transcription polymerase chain reaction testing for COVID-19 on throat swabs; (3) a thin-section chest CT scan showing any evidence of pneumonia; and (4) patients admitted for antiviral treatment. Patients with mechanical ventilation in the course were excluded because of the severe moving artifacts in chest CT images. Figure 1 shows the pathway of patient inclusion. After admission, clinical data including demographics, comorbidities, and symptoms of patients and laboratory tests were collected. The data in source documents were confirmed independently by at least two researchers.

### CT Image Acquisition

Subjects were referred to the radiology department based on the algorithm suggested by evidence presented by the World Health Organization (39). All patients underwent chest CT scans by a 64-slice CT scanner (Siemens Definition AS + 128, Forchheim, Germany). All patients were scanned in the supine position from the lung apex to the diaphragm during end-inspiration. To reduce breathing artifacts, patients were instructed on breath-holding. No contrast agent was administered. CT acquisition was executed as follows: tube voltage, 120 kV; tube current, auto mAs; pitch, 1.2; rotation time, 0.5 s; field of view, 330 × 330 mm. Lung images were reconstructed at a slice thickness of 1.0 or 1.25 mm using I50 medium sharp algorithm. Lung window level and window width were set as −530 to 430 Hounsfield units (HU) and 1,400–1,600 HU, respectively.

### Quantitative CT Analysis

**Figure 2:**
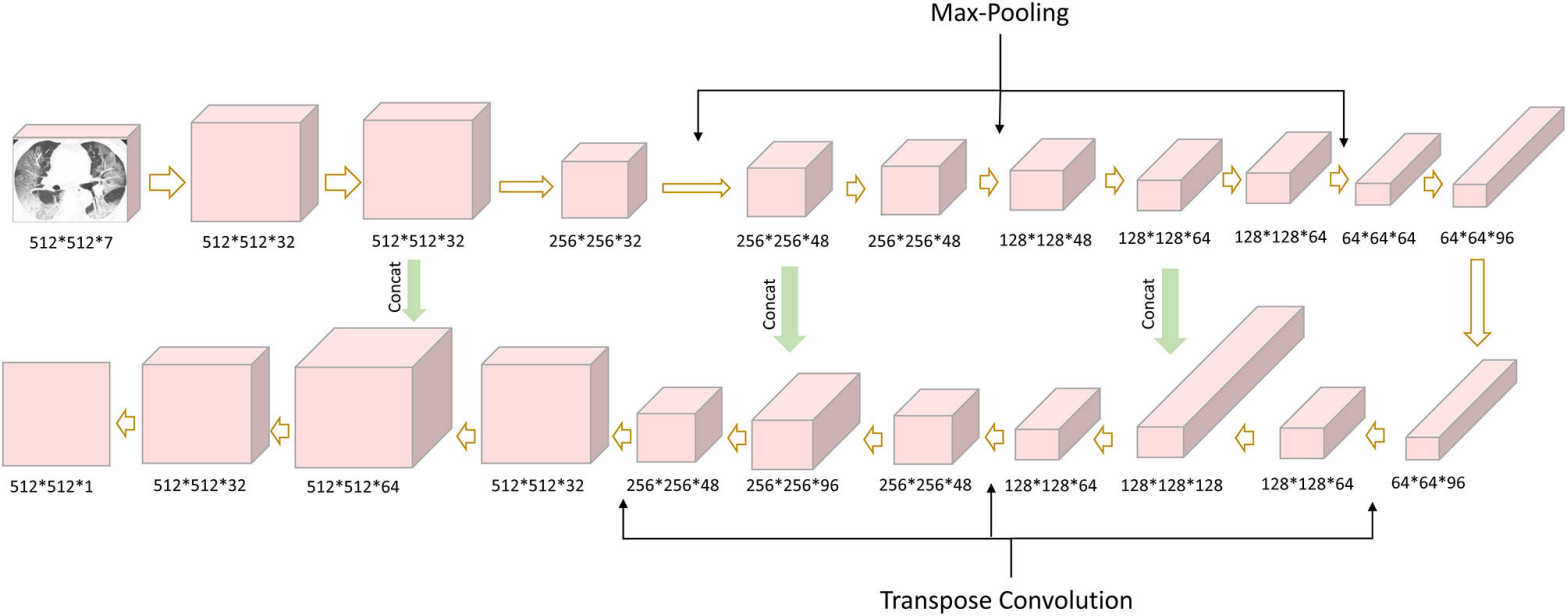
COVID-Lesion Net structure for pneumonia detection and segmentation.

The quantification analysis of CT images was performed by a care.ai Intelligent Multi-disciplinary Imaging Diagnosis Platform Intelligent Evaluation System of Chest CT for COVID-19 (YT-CT-Lung, YITU Healthcare Technology Co., Ltd., China). This system was constructed using a combination of U-net and fully convolutional networks (40, 41), which consists of three different network components: (1) 12 convolutional segments, which included convolutional layer (Conv2d), batch normalization layer, and an activation layer; (2) three max-pooling layers for down-sampling; and (3) three transpose convolutional layer for up-sampling (Figure 2). The development of the COVID-Lesion Net has been described in a previous study (42).

**Figure 3:**
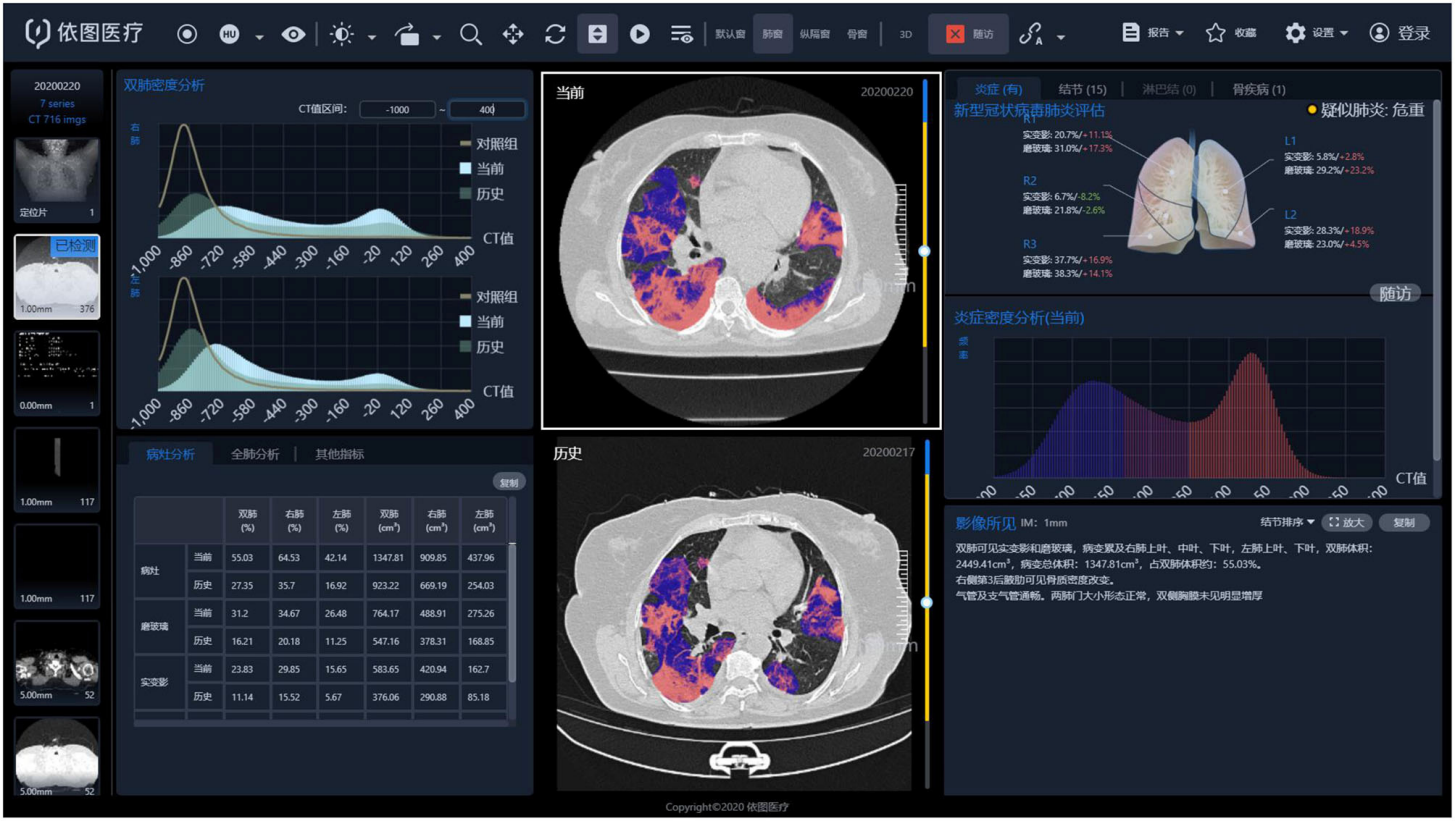
CT image quantization and analysis with artificial intelligence system.

Subsequently, by thresholding on CT values in the pneumonia lesions using two-dimensional neural network for classification, two quantitative features were generated, that is, ground-glass opacities (GGOs) with value ranges of −600 to −500 HU and consolidation with density ranges of −250 to 60 HU (43). A quantitative analysis of pneumonia lesions, GGO, and consolidation was performed based on the segmentation results, including the percentage of pneumonia volume (PPV), GGO volume (PGV), and consolidation volume (PCV) in both lungs, left lung, right lung, and five lobes (Figure 3). It took about 10 s to calculate the various CT parameters. All the image segmentations were reviewed independently and assessed by two radiologists (with 10 and 20 years of experience in thoracic imaging), and discrepancies were resolved by consensus.

### Definition of Endpoint

We defined the severity of COVID-19 according to the newest COVID-19 guidelines released by the National Health Commission of China (44). We defined critical illness as a composite of admission to intensive care unit, respiratory failure requiring mechanical ventilation, shock, or death.

### Statistical Analysis

Categorical variables were expressed as counts and percentages, whereas continuous variables are shown as median and interquartile range. Continuous variables were compared using *t*-test or Mann–Whitney *U*-test, and categorical variables were compared using χ^2^-test or Fisher exact test. CT features were compared using *t*-test or Mann–Whitney *U*-test. The optimal cutoff value for discriminating critical and non-critical COVID-19 patients was determined by using receiver operating characteristic analysis and maximizing the Youden index. The correlations between total pneumonia volume and laboratory variables were assessed by Pearson correlation analysis. *P* < 0.05 was considered significant. All statistical analyses were conducted by IBM SPSS version 22.0 (Chicago, IL, USA).

## Results

### Clinical Characteristics of Patients

This study finally included 140 patients, excluding 37 patients without evidence of COVID-19 lesions, and 12 had severe moving artifacts in chest CT images. Table 1 demonstrates the clinical characteristics of patients. Among the 140 patients with COVID-19, 68 (48.6%) were moderate, 40 (28.6%) were severe, and 32 (22.9%) were critical (including 12 deaths). The mean age of all patients was 58.6 ± 13.8 years (range, 25–86 years), and 85 patients (60.7%) were male. Fever (81.4%) was the most common symptom, followed by dry cough (76.4%), shortness of breath (55.0%), and fatigue (47.1%). Sixty-one patients (43.6%) had at least one comorbidity, with hypertension (30.0%) being the most common, followed by diabetes (16.4%) and cardiovascular disease (11.4%).

**Table 1:** Clinical characteristics of patients.

**Characteristics**	
**Age (years)**	58.6 ± 13.7
**Sex**	
Male	85 (60.7)
Female	55 (39.3)
**Comorbidities**	
COPD	6 (4.3)
Cardiovascular disease	16 (11.4)
Hypertension	42 (30.0)
Diabetes	23 (16.4)
**Symptoms**	
Fever	114 (81.4)
Dry cough	107 (76.4)
Shortness of breath	77 (55.0)
Diarrhea	9 (6.4)
Anorexia	32 (22.9)
Fatigue	66 (47.1)
**Disease severity**	
Moderate	68
Severe	40
Critical	32
**Laboratory findings**	
WBCs (×10^9^/L)	6.3 ± 3.7
Neutrophil (×10^9^/L)	4.9 ± 3.7
Neutrophil (%)	69.8 ± 15.0
Lymphocyte (×10^9^/L)	1.2 ± 0.6
Lymphocyte (%)	21.6 ± 11.6
Eosinophil (×10^9^/L)	0.08 ± 0.08
Eosinophil (%)	1.4 ± 1.5
Monocyte (×10^9^/L)	0.4 ± 0.2
Monocyte (%)	7.2 ± 3.1
Hemoglobin (g/L)	124.2 ± 22.3
Platelet (g/L)	220.1 ± 75.7
Fibrinogen (g/L)	4.0 ± 2.2
d-Dimer (μg/mL)	0.8 ± 1.4
hs-CRP (mg/L)	19.1 ± 26.1
ALT (U/L)	38.3 ± 49.0
AST (U/L)	31.6 ± 28.4
TBIL (μmol/L)	12.5 ± 9.1
DBIL (μmol/L)	5.5 ± 18.3
ALP (U/L)	63.8 ± 26.8
Myohemoglobin (μg/L)	45.3 ± 37.5
CK (ng/mL)	75.7 ± 91.9
LDH (U/L)	260.7 ± 121.0
PLT (ng/mL)	0.3 ± 1.6
ESR (s)	38.4 ± 30.1
NT-proBNP (pg/mL)	318.9 ± 435.6
Scr (μmol/L)	71.8 ± 18.4
BUN (mmol/L)	5.3 ± 2.8
NLR	5.8 ± 8.8

*Data were mean ± standard deviation (SD) or number*.

*COPD, chronic obstructive pulmonary disease; WBC, white blood cells; hs-CRP, high-sensitivity C-reactive protein; ALT, alanine transaminase; AST, aspartate aminotransferase; TBIL, total bilirubin; DBIL, direct bilirubin; ALP, alkaline phosphatase; LDH, lactate dehydrogenase; CK, creatine kinase; PLT, procalcitonin; ESR, erythrocyte sedimentation rate; NT-proBNP, N-terminal brain natriuretic peptide precursor; Scr, serum creatinine; BUN, urea nitrogen; NLR, neutrophil-to-lymphocyte ratio*.

### CT Features and Laboratory Variables in the Critical and Non-Critical Groups

**Figure 4:**
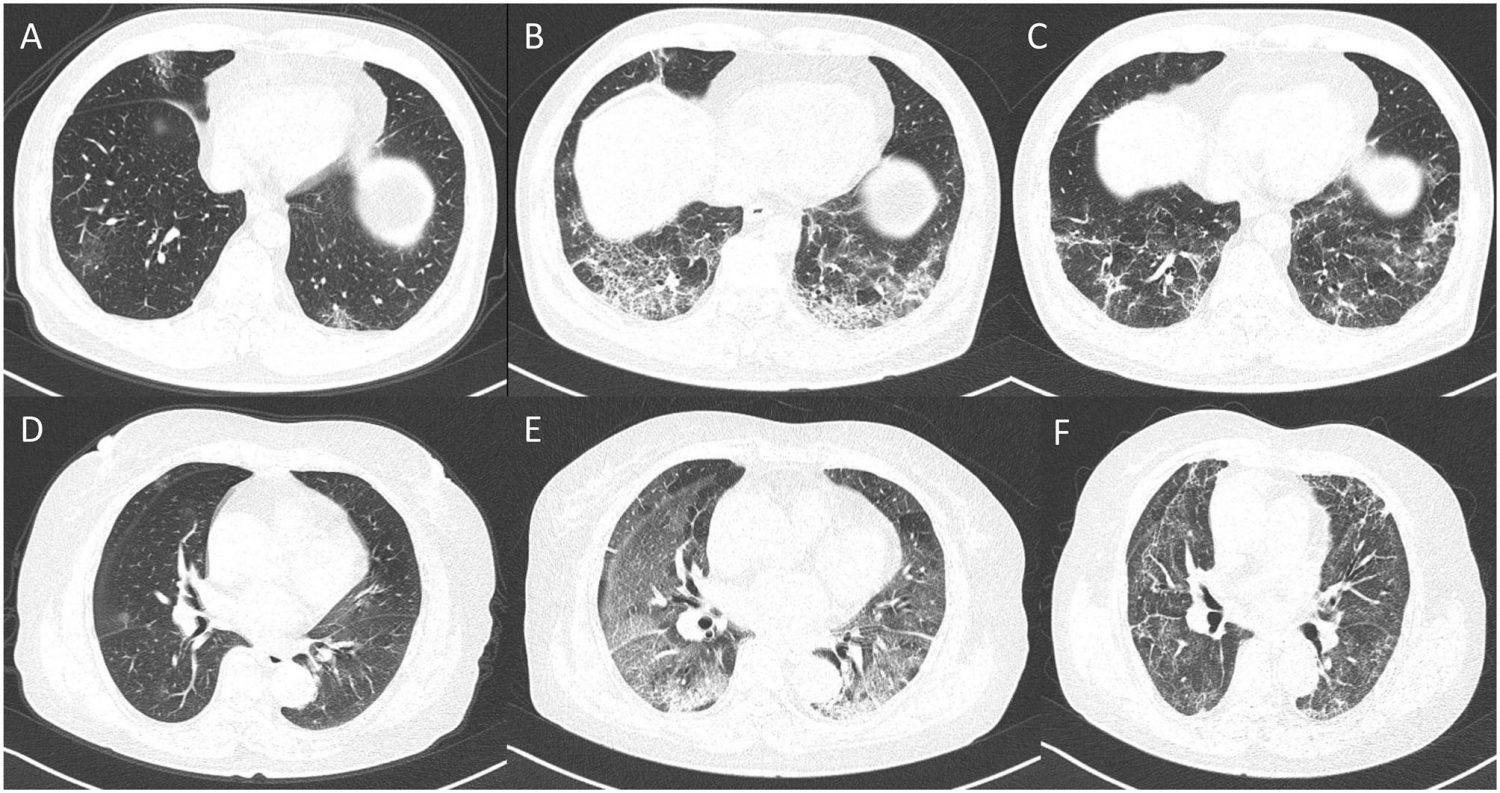
Dynamic changes in lung lesions in two representative cases with COVID-19 pneumonia. A 65-year-old man: **(A)** scan obtained on day 2; multiple patchy GGOs were shown in the middle lobe and both lower lobes; the PPV, PGV, and PCV were 1.14, 1.07, and 0.07%, respectively. **(B)** Scan obtained on day 7 showed extensive GGOs with consolidation and crazy-paving pattern in the both lower lobes; the PPV, PGV, and PCV increased to 14.24, 11.38, and 2.86%, respectively. **(C)** Scan obtained on day 21 showed obvious absorption of abnormalities with fibrotic-like appearances; the PPV, PGV, and PCV were 7.73, 7.12, and 0.61%, respectively. A 67-year-old woman: **(D)** Scan obtained on day 2, small nodule of GGO was shown in the right lung; the PPV, PGV, and PCV were 0.19, 0.16, and 0.03%, respectively. **(E)** Scan obtained on day 10 showed extensive GGOs in both lungs with consolidation in the lower lobes; the PPV, PGV, and PCV increased to 48.67, 38.23, and 10.44, respectively. **(F)** Scan obtained on day 56 showed obvious absorption of GGOs and consolidation with fibrotic-like appearances; the PPV, PGV, and PCV were 39.47, 33.77, and 5.70%, respectively.

Table 2 shows the difference of clinical and laboratory variables between the non-critical and critical groups. There were a total of 28 laboratory variables for the two groups. The median time from symptom onset to CT examination among the moderately, severely, and critically ill groups was 11, 10, and 10 days, respectively (*p* = 0.250). Comparison of quantitative CT features between the non-critical and critical groups is depicted in Table 3. There were 24 CT features for the two groups. The PPV, PGV, and PCV in the left lung, right lung, both lungs, and five lobes were significantly higher in the critical group than the non-critical group (all *p* < 0.001). Figure 4 shows temporal changes in lung lesions in two representative cases with COVID-19 pneumonia.

**Table 2:** Comparison of clinical and laboratory variables between the non-critical and critical groups.

**Characteristics**	**Non-critical (*n* = 108)**	**Critical** ** (*n* = 32)**	***P*-value**
**Age (years)**	57.6 ± 14.3	61.9 ± 11.5	0.093
**Sex**			
Male	61 (56.5)	24 (75.0)	0.060
Female	47 (43.5)	8 (25.0)	
**Comorbidities**			
COPD	6 (5.6)	0	0.336
Cardiovascular disease	14 (13.0)	2 (6.3)	0.364
Hypertension	30 (27.8)	12 (37.5)	0.292
Diabetes	16 (14.8)	7 (21.9)	0.344
**Symptoms**			
Fever	82 (75.9)	32 (100)	**0.002**
Dry cough	76 (70.4)	31 (96.9)	**0.002**
Shortness of breath	46 (42.6)	31 (96.9)	** <0.001**
Diarrhea	6 (5.6)	3 (9.4)	0.427
Anorexia	28 (25.9)	4 (12.5)	0.112
Fatigue	47 (43.5)	19 (59.4)	0.115
**Laboratory findings**			
WBCs (×10^9^/L)	5.7 ± 2.9	8.4 ± 5.6	**0.010**
Neutrophil (×10^9^/L)	4.2 ± 2.8	7.4 ± 5.1	** <0.001**
Neutrophil (%)	66.6 ± 14.7	81.2 ± 9.7	** <0.001**
Lymphocyte (×10^9^/L)	1.3 ± 0.6	0.9 ± 0.5	**0.001**
Lymphocyte (%)	24.4 ± 11.1	11.7 ± 6.5	** <0.001**
Eosinophil (×10^9^/L)	0.08 ± 0.07	0.06 ± 0.09	0.061
Eosinophil (%)	1.5 ± 1.5	0.7 ± 1.1	**0.002**
Monocyte (×10^9^/L)	0.4 ± 0.2	0.5 ± 0.3	0.183
Monocyte (%)	7.4 ± 3.1	6.1 ± 2.8	0.083
Hemoglobin (g/L)	124.9 ± 20.4	121.7 ± 28.8	0.694
Platelet (g/L)	225.5 ± 74.2	200.3 ± 79.4	0.090
Fibrinogen (g/L)	3.8 ± 2.2	5.0 ± 1.5	**0.001**
d-Dimer (μg/mL)	0.6 ± 0.9	1.6 ± 2.4	0.055
hs-CRP (mg/L)	14.9 ± 18.0	46.0 ± 47.5	** <0.001**
ALT (U/L)	33.9 ± 45.4	56.6 ± 59.5	**0.004**
AST (U/L)	26.9 ± 23.8	53.2 ± 37.4	** <0.001**
TBIL (μmol/L)	12.5 ± 9.8	12.6 ± 5.8	0.435
DBIL (μmol/L)	5.7 ± 20.3	4.6 ± 2.5	0.054
ALP (U/L)	63.5 ± 26.2	65.7 ± 31.4	0.719
Myohemoglobin (μg/L)	35.2 ± 25.9	79.5 ± 62.7	0.059
CK (ng/mL)	66.3 ± 91.7	107.4 ± 92.8	0.053
LDH (U/L)	225.7 ± 112.3	378.8 ± 147.3	** <0.001**
PLT (ng/mL)	0.3 ± 1.8	0.2 ± 0.3	0.270
ESR (s)	35.1 ± 31.9	49.4 ± 23.1	0.423
NT-proBNP (pg/mL)	315.5 ± 418.0	330.5 ± 491.7	0.850
Scr (μmol/L)	69.2 ± 16.5	81.2 ± 22.1	**0.012**
BUN (mmol/L)	4.6 ± 1.6	7.7 ± 4.5	** <0.001**
NLR	4.8 ± 8.7	9.3 ± 8.3	** <0.001**

*Data were mean ± SD or number (percentage). P-values were calculated by t-test, Mann–Whitney U-test, χ^2^-test, or Fisher exact test, as appropriate*.

*COPD, chronic obstructive pulmonary disease; WBCs, white blood cells; hs-CRP, high-sensitivity C-reactive protein; ALT, alanine transaminase; AST, aspartate aminotransferase; TBIL, total bilirubin; DBIL, direct bilirubin; ALP, alkaline phosphatase; LDH, lactate dehydrogenase; CK, creatine kinase; PLT, procalcitonin; ESR, erythrocyte sedimentation rate; NT-proBNP, N-terminal brain natriuretic peptide precursor; Scr, serum creatinine; BUN, urea nitrogen; NLR, neutrophil-to-lymphocyte ratio. Bold values indicate P < 0.05*.

**Table 3:** Comparison of quantitative CT features between non-critical and critically ill groups.

**CT features**	**Non-critical (*n* = 108)**	**Critically ill (*n* = 32)**	***P*-value**
**PPV**			
PPV in both lungs	12.4 (41.8)	41.8 (20.9)	** <0.001**
PPV in the left lung	11.7 (14.4)	39.2 (22.1)	** <0.001**
PPV in the upper lobe of left lung	8.8 (13.1)	34.7 (21.7)	** <0.001**
PPV in the lower lobe of left lung	16.4 (20.0)	46.0 (24.9)	** <0.001**
PPV in the right lung	13.2 (15.8)	44.1 (21.9)	** <0.001**
PPV in the upper lobe of right lung	11.1 (15.8)	41.9 (24.0)	** <0.001**
PPV in the middle lobe of right lung	7.5 (13.1)	31.4 (22.6)	** <0.001**
PPV in the lower lobe of right lung	18.2 (21.6)	51.4 (25.0)	** <0.001**
**PGV**			
PGV in both lungs	10.0 (11.4)	32.4 (16.4)	** <0.001**
PGV in the left lung	9.5 (11.7)	30.1(16.8)	** <0.001**
PGV in the upper lobe of left lung	7.6 (11.2)	27.9 (16.8)	** <0.001**
PGV in the lower lobe of left lung	12.5 (15.2)	33.2 (18.4)	** <0.001**
PGV in the right lung	10.7 (12.8)	34.3 (17.5)	** <0.001**
PGV in the upper lobe of right lung	9.5 (13.4)	33.7 (20.1)	** <0.001**
PGV in the middle lobe of right lung	6.7 (11.6)	28.0 (20.3)	** <0.001**
PGV in the lower lobe of right lung	13.8 (16.1)	37.5 (18.1)	** <0.001**
**PCV**			
PCV in both lungs	2.4 (3.3)	9.4 (7.8)	** <0.001**
PCV in the left lung	2.3 (3.4)	9.2 (9.0)	** <0.001**
PCV in the upper lobe of left lung	1.3 (2.7)	6.8 (7.7)	** <0.001**
PCV in the lower lobe of left lung	3.9 (5.9)	12.8 (11.9)	** <0.001**
PCV in the right lung	2.5 (3.7)	9.8 (7.8)	** <0.001**
PCV in the upper lobe of right lung	1.6 (3.0)	8.2 (7.6)	** <0.001**
PCV in the middle lobe of right lung	0.8 (1.8)	3.4 (2.9)	** <0.001**
PCV in the lower lobe of right lung	4.4 (7.0)	13.9 (11.2)	** <0.001**

*PPV, percentage of pneumonia volume; PGV, percentage of ground-glass opacity volume; PCV, percentage of consolidation volume. Bold values indicate P < 0.05*.

### Associations of CT Features With Critical Illness

**Figure 5:**
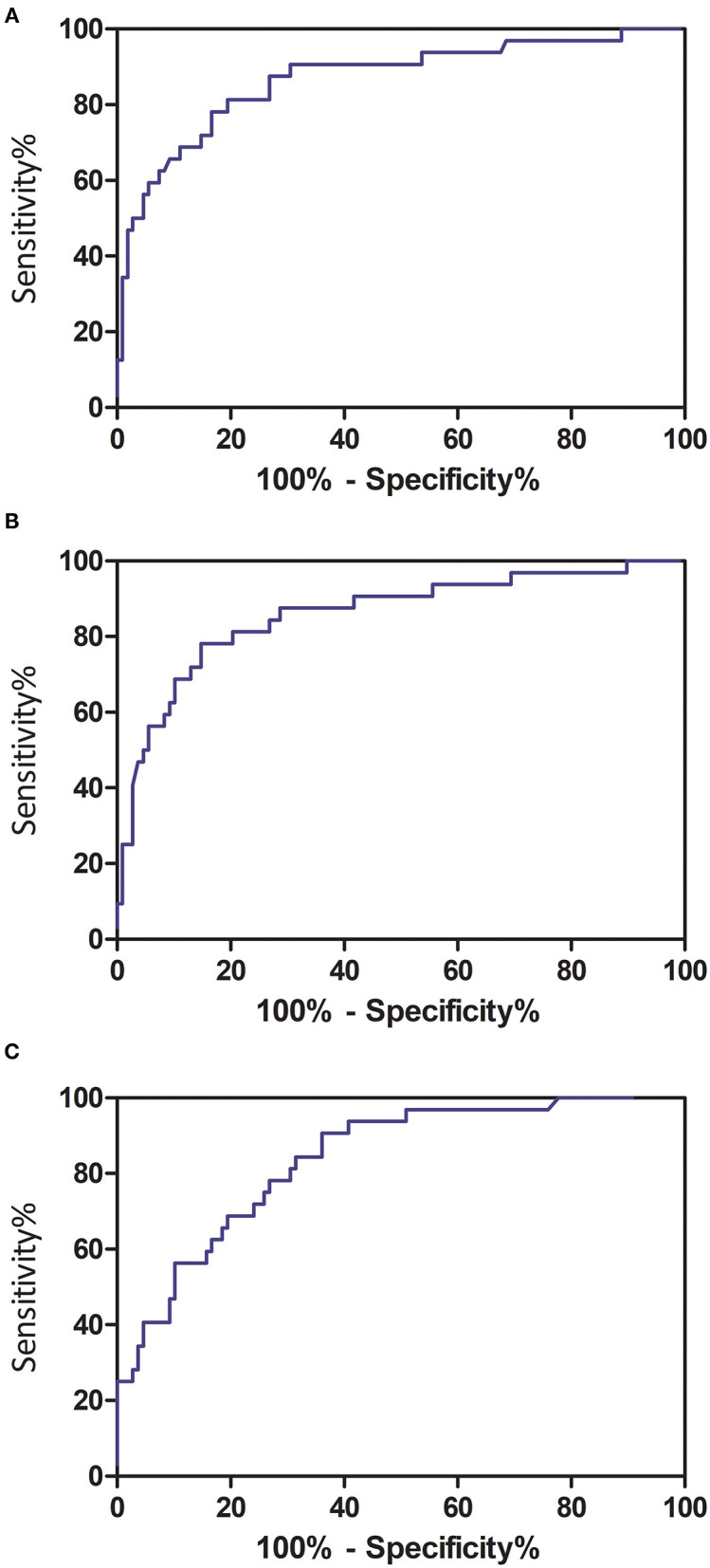
Receiver operating characteristic curve analyses of quantitative CT features in predicting critical illness among COVID-19 patients. **(A)** Total pneumonia volume in both lungs; **(B)** ground-glass opacity volume in both lungs; and **(C)** consolidation volume in both lungs.

The optimal cutoff value of PPV in both lungs was 22.6%, yielding an area under the curve (AUC) of 0.868 [95% confidence interval (CI) = 0.791–0.946], sensitivity of 81.3% (95% CI = 63.6–92.8%), and specificity of 80.6% (95% CI = 71.8–87.5%) in predicting the occurrence of critical illness of COVID-19 patients (Figure 5A). The optimal cutoff value of PGV in both lungs was 21.3%, obtaining an AUC of 0.858 (95% CI = 0.778–0.938), sensitivity of 78.1% (95% CI = 60.0–90.7%), and specificity of 85.2% (95% CI = 77.1–91.3%) in predicting the occurrence of critical illness of COVID-19 patients (Figure 5B). The optimal cutoff value of PCV in both lungs was 1.8%, achieving an AUC of 0.838 (95% CI = 0.764–0.912), sensitivity of 90.6% (95% CI = 75.0–98.0%), and specificity of 63.9% (95% CI = 54.1–72.9%) in predicting the occurrence of critical illness of COVID-19 patients (Figure 5C).

### Correlations Between Total Pneumonia Volume and Laboratory Variables

**Figure 6:**
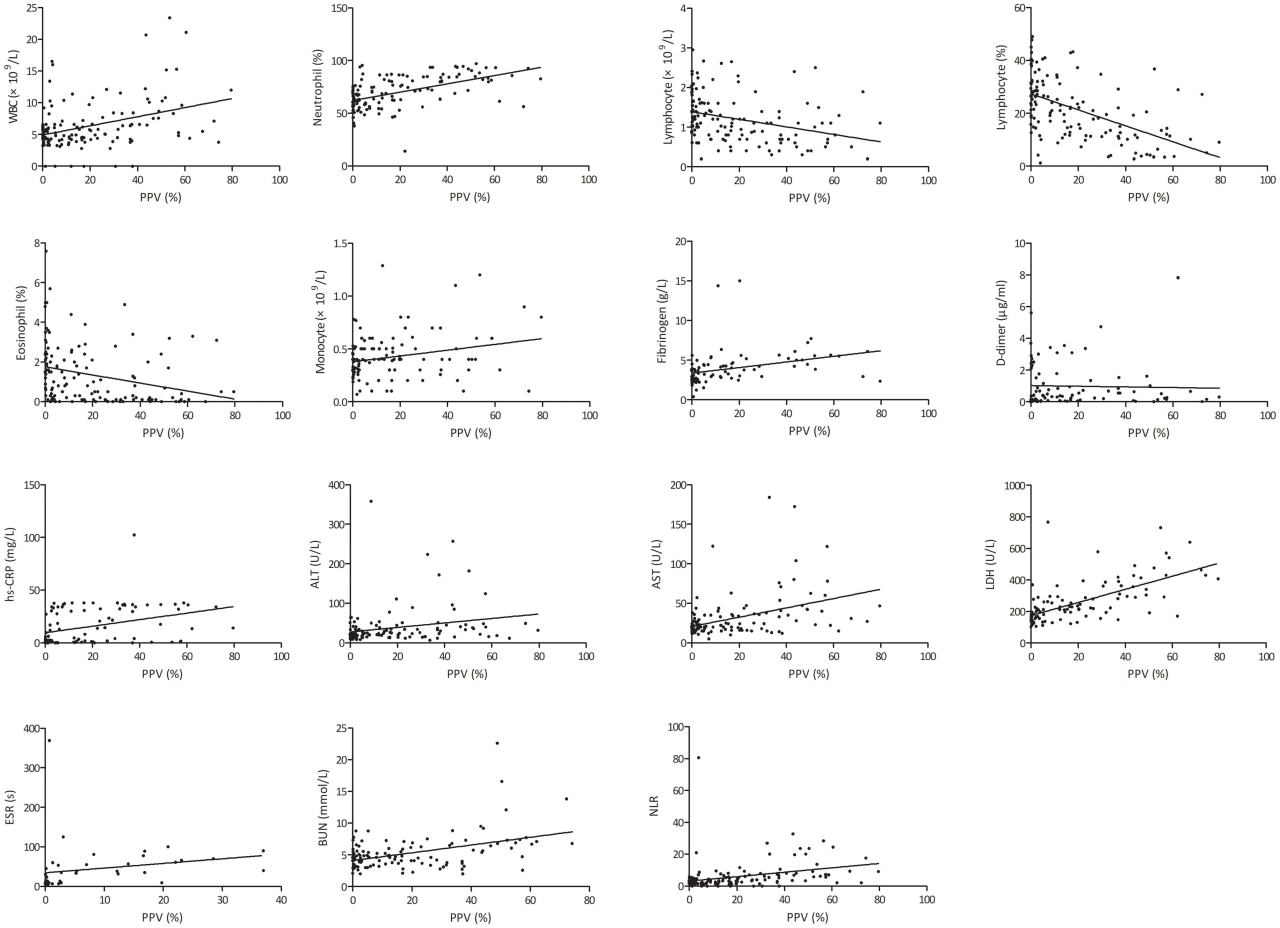
Correlation of total pneumonia volume with different clinical parameters. hs-CRP, high-sensitivity C-reactive protein; ALT, alanine transaminase; AST, aspartate aminotransferase; LDH, lactic dehydrogenase; NLR, neutrophil-to-lymphocyte ratio; PPV, percentage of pneumonia volume.

The PPV was positively correlated with white blood cell (WBC) count (*r* = 0.379, *p* < 0.001), neutrophil percentage (*r* = 0.535, *p* < 0.001), monocyte count (*r* = 0.244, *p* = 0.010), neutrophil-to-lymphocyte ratio (NLR) (*r* = 0.318, *p* < 0.001), erythrocyte sedimentation rate (ESR) (*r* = 0.567, *p* < 0.001), fibrinogen (*r* = 0.324, *p* = 0.003), d-Dimer (*r* = 0.444, *p* < 0.001), high-sensitivity C-reactive protein (hs-CRP) (*r* = 0.495, *p* < 0.001), alanine transaminase (*r* = 0.231, *p* = 0.012), aspartate aminotransferase (*r* = 0.410, *p* < 0.001), lactic dehydrogenase (LDH) (*r* = 0.644, *p* < 0.001), and blood urea nitrogen (*r* = 0.439, *p* < 0.001), whereas the PPV was negatively correlated with lymphocyte count (*r* = −0.323, *p* < 0.001), lymphocyte percentage (*r* = −0.523, *p* < 0.001), and eosinophil percentage (*r* = −0.285, *p* = 0.001) (Figure 6).

## Discussion

This current study showed that artificial intelligence (AI)–derived quantitative CT features could predict the deterioration to critical illness in patients with COVID-19, in particular, the pneumonia volume percentage. Also, these CT features were correlated with laboratory variables reflecting systemic inflammation, immune state, and multiple organ functions.

Lung CT can provide useful additional information in the detection, diagnosis, and follow-up of COVID-19 pneumonia (5). However, CT images are usually visually interpreted by radiologists with diverse levels of experience, which is subjective with large variability that is unable to quantitatively assess the disease severity and is also time-consuming and labor-intensive. Previous studies have shown that quantitative CT is comparable or superior to visual CT score in assessment of the severity of COVID-19 (28, 45, 46). Recently, several studies have used quantitative CT to predict clinical outcomes *via* AI software in patients with COVID-19 (43, 47, 48). Liu et al. found that quantitative CT features on days 0 and 4 as well as changes from days 0 to 4 could predict the progression to severe illness in COVID-19 patients, which outperformed the acute physiology and chronic health evaluation II score, NLR, and d-Dimer (43). Homayounieh et al. found that despite a high frequency of motion artifacts, quantitative features of pulmonary opacities from chest CT can help stratify patients with favorable and adverse outcomes (47). Salvatore et al. demonstrated that quantification of the consolidation, emphysema, and residual healthy lung parenchyma on chest CT images were independent predictors of outcome in patients with COVID-19 pneumonia (48). Our study demonstrated that quantitative CT measurements at admission could accurately predict adverse outcomes in COVID-19 patients. The total lesion volume had the best performance of assessing the severity of COVID-19, which was in agreement with a previous study (49). With the advance of image data-mining tools, radiomics and deep learning play a crucial role in the prediction of severity, prognosis, or outcome of patients with COVID-19 (7–34). The COVID-19 lesion images contained high-level features that can effectively represent morphological appearances and heterogeneous information. The handcrafted and learning features derived from CT images can be integrated into clinical and laboratory variables to form a combined model with more favorable performance. These models provided physicians with an important tool for improving the clinical care of patients with the worse disease outcomes.

The extent of GGO and consolidation can evaluate the disease severity of COVID-19 (50). As viruses spread *via* the respiratory mucosa and also infect other cells, they induce a cytokine storm and a series of immune responses that cause changes in peripheral blood and immune cells (51). Coronaviruses invade the lungs, as well as the blood system, digestive system, and circulatory system (52). Therefore, the disease severity assessed by chest CT may correlate with laboratory inflammatory and immune biomarkers (53). The severity of lymphopenia and infection correlated with the severity of COVID-19 (54). Zhang et al. found that CT score had positive associations with inflammatory mediators, including WBC count, neutrophil count, prothrombin time, d-Dimer, CRP, ESR, procalcitonin, serum ferritin, IL-6, and IL-10, but a negative association with lymphocyte count (51). Another study revealed dynamic correlation between CT score and laboratory parameters, which showed that CT score at an early stage was correlated with neutrophil count, whereas CT score at progressive stage was correlated with neutrophil count, WBC count, hs-CRP, procalcitonin, and LDH (55). The correlation between laboratory, clinical data, and CT quantitative features has also been shown in several studies (49, 56). Kang et al. observed that histogram features were significantly correlated with National Early Warning Score, neutrophil percentage, procalcitonin, acute respiratory distress syndrome, and extracorporeal membrane oxygenation and negatively correlated with lymphocyte percentage and lymphocyte count (56). Sun et al. demonstrated that CT quantitative parameters were significantly correlated with inflammatory markers, including neutrophil percentage, lymphocyte count, lymphocyte percentage, hs-CRP, and procalcitonin (49). These findings may prove the reliability of quantitative CT in assessment of disease severity at the level of biology.

This study has some limitations. First is the retrospective nature of this study with small sample size. Therefore, a large cohort is needed to validate the role of AI-derived CT features in assessment of prognosis of COVID-19 patients. Second, the effect of anti–COVID-19 treatment on prognosis was not considered because no specific strategies have been used in the treatment of COVID-19 except for supportive care until now. Third, CT-based radiomics or deep learning and follow-up CT scan may provide more prognostic information.

Our study illustrated that AI-derived CT features were correlated with laboratory variables reflecting systemic inflammation, immune state, and multiple organ functions (e.g., coagulation, liver, and renal functions). Thus, CT quantitative analysis might be an effective and important method for assessing the severity of COVID-19 and may provide additional guidance for planning clinical treatment strategies. This technique can be used in routine practice. Large-scale prospective studies in the future are warranted to confirm the CT features in predicting the occurrence of critical illness in COVID-19 patients.

## Data Availability Statement

The original contributions presented in the study are included in the article/supplementary material, further inquiries can be directed to the corresponding author/s.

## Ethics Statement

The studies involving human participants were reviewed and approved by First Affiliated Hospital of Guangzhou Medical University. The ethics committee waived the requirement of written informed consent for participation.

## Author Contributions

BP, HL, QL, and PW: literature search. BP, HL, QL, XZ, PW, and TX: study design. BP, HL, QL, and XZ: data collection. TX, PW, WL, JL, LL, CO, JM, and SL: data analysis. FZ, XW, and JX: data interpretation. BP, HL, QL, PW, and XZ: writing. All authors manuscript review and approval.

## Conflict of Interest

The authors declare that the research was conducted in the absence of any commercial or financial relationships that could be construed as a potential conflict of interest.
